# Assessment of the Diuretic Properties of Rice Bean Accessions Using a Mouse Model and Identification of Active Polyphenolic Compounds

**DOI:** 10.3390/nu16111603

**Published:** 2024-05-24

**Authors:** Dan Gong, Bin Zhang, Yang Yao, Suhua Wang, Tao Xiong, Lixia Wang

**Affiliations:** 1 Key Laboratory of Grain Crop Genetic Resources Evaluation and Utilization, Institute of Crop Sciences, Chinese Academy of Agricultural Sciences, Beijing 100081, China; gongdan0129@126.com (D.G.); yaoyang@caas.cn (Y.Y.); wangsuhua@caas.cn (S.W.); 2 College of Life Sciences, Yangtze University, Jingzhou 434022, China; 15738152677@163.com (B.Z.); xiongtao@hotmail.com (T.X.)

**Keywords:** rice beans, diuretic effect, urinary output, water intake, weight loss, polyphenols

## Abstract

Rice bean [*Vigna umbellata* (Thunb.) Ohwi and Ohashi], an annual legume in the genus *Vigna*, is a promising crop suitable for cultivation in a changing climate to ensure food security. It is also a medicinal plant widely used in traditional Chinese medicine; however, little is known about the medicinal compounds in rice bean. In this study, we assessed the diuretic effect of rice bean extracts on mice as well as its relationship with the contents of eight secondary metabolites in seeds. Mice gavaged with rice bean extracts from yellow and black seeds had higher urinary output (5.44–5.47 g) and water intake (5.8–6.3 g) values than mice gavaged with rice bean extracts from red seeds. Correlation analyses revealed significant negative correlations between urine output and gallic acid (R = −0.70) and genistein (R = −0.75) concentrations, suggesting that these two polyphenols negatively regulate diuresis. There were no obvious relationships between mice diuresis-related indices (urine output, water intake, and weight loss) and rutin or catechin contents, although the concentrations of both of these polyphenols in rice bean seeds were higher than the concentrations of the other six secondary metabolites. Our study findings may be useful for future research on the diuretic effects of rice bean, but they should be confirmed on the basis of systematic medical trials.

## 1. Introduction

Rice bean [*Vigna umbellata* (Thunb.) Ohwi and Ohashi] is a warm-season annual species in the genus *Vigna*. It originated in South and Southeast Asia and was probably first domesticated in the Indo-China region [1,2]. It is commonly cultivated in South, Southeast, and East Asia and was recently introduced in the Americas, Australia, and Africa [3,4]. Rice bean, which grows well in a hot humid climate [5], can thrive in various soils, including barren, sandy loam, clay, and acid soils [6,7]. It is often used as the donor parent for interspecific hybridizations [8] because of its strong resistance to pests [9,10,11], diseases [12], and drought [13,14], as well as its high grain yield and large biomass [4]. Rice bean is usually cultivated as part of an intercropping or rotation system because of its ability to fix nitrogen [6,15]. There has been relatively little genetic improvement of rice bean. Moreover, because of their prostrate growth and indeterminate flowering, most rice bean cultivars must be harvested by hand [1]. The associated labor costs have been an impediment to the large-scale cultivation of rice bean [16].

The health benefits of rice bean are due to its high nutritional quality [17,18] and the fact its seed is rich in protein, carbohydrates, vitamins, and minerals but has a low fat content [19,20,21]. In addition, most of the above-ground rice bean plant tissues, including the leaves, flowers, shoots, and young pods, can be consumed as food (i.e., vegetables) or used as silage [6]. According to Shennong’s Classic of Materia Medica, rice bean has detoxifying, anti-inflammatory, and diuretic effects [22]. Modern pharmacological research showed that rice bean has excellent antioxidant, hypoglycemic, and hypolipidemic properties [23]. Earlier research suggested that dry rice bean seeds are rich in various bioactive substances, including polyphenols, tannins, and saponins [24]; these secondary metabolites might be the main factors underlying the medicinal properties (i.e., antioxidant and diuretic effects) [25]. Of these diverse secondary metabolites, it is unclear which one is most related to diuresis. Thus, the main objective of this study was to identify and characterize the main substances, especially secondary metabolites, responsible for the diuretic effect of rice bean. We evaluated the diuretic effect of rice bean on mice by conducting a gavage test using the supernatant of boiled rice bean accessions. We also determined the concentrations of eight secondary metabolites in the seeds of each accession. On the basis of the relationship between the diuresis-related indices and secondary metabolite contents, the probable bioactive components mediating the diuretic effect were determined. Our results may be relevant to future studies on the mechanism underlying diuresis as well as the development of health products using rice bean, thereby increasing the economic benefits of this crop.

## 2. Materials and Methods

### 2.1. Materials

Animals: Mice (approximately 15 g each) were purchased from Jingzhou Huaqiao Biological Co., Ltd. (Jingzhou, China).

Seeds: Nine rice bean accessions that differed in terms of seed color and weight were used for the analysis of eight secondary metabolite contents as well as for animal experiments. Two adzuki bean accessions and one mung bean accession were used as checks. All seeds were harvested in 2022 from the Sanya Experimental Base (18.38° N, 109.21° E) of the Institute of Crop Science, Chinese Academy of Agricultural Sciences (Table 1).

### 2.2. Preparation of Gastric Infusions

For each accession, 200 g seeds were ground to a powder using a grinder and then sealed and stored at 4 °C. During the experimental period, 10 g of ground material was added to 100 mL H_2_O in the morning, boiled for 30 min, and then centrifuged (1000 rpm for 5 min at 25 °C). The supernatant was filtered through gauze and retained until used.

### 2.3. Gavage and Data Collection

For 1 week, mice were provided free access to food and drinking water to observe the stability of their urine output. The mice were then randomly divided into 13 groups (one mock control group and 12 treatment groups, with five mice per group) for gavage treatments. The 12 treatments, which were named according to accession codes, were as follows: FD28, FD76, FD113, FD297, SY02, SY03, SY06, R20, and R22 (rice bean accessions), PH2013-161 and THM2011-28 (adzuki bean accessions), and Zhonglv 27 (mung bean accession). The mice in different treatment groups were gavaged with 0.5 mL supernatant, whereas the mice in the mock control group were gavaged with 0. 5 mL water. Before gavage, the mice were fasted without water for 12 h. Each group of mice and the water were weighed at 9:00 a.m. the next day. After gavage, the mice were fed normally, and the mice and water were weighed again at 5:00 p.m. The experiment lasted for 7 days, during which the urine of each group of mice was collected on filter paper. The weight of the filter paper was measured hourly, and after 8 h, the urine volume of the mice in each group was calculated. The difference in weight between the morning and evening was used to calculate the changes in the weight of mice and the amount of water intake.

### 2.4. Analysis of Polyphenols in Legume Seeds

Legume seed extracts were screened for eight polyphenols (gallic acid, chlorogenic acid, catechins, caffeic acid, rutin, vitexin, isovitexin, and genistein). All compounds were examined using a high-performance liquid chromatography system (LC-20ADXR; Shimadzu, Tokyo, Japan). The chromatographic analysis was performed using a BEH C_18_ column (100 mm × 2.1 mm, 1.7 μm) (Waters, Milford, MA, USA) at 25 °C with an injection volume of 20 µL. For the gradient elution, acetonitrile (A) and 0.1% formic acid–water (B) were used at a flow rate of 0.3 mL/min. The gradient elution program was as follows: 90% B at 0–5 min, 90–78% B at 5–27.5 min, 78% B at 27.5–38 min, 78–90% B at 38–43 min, and 90% B at 43–55 min. Eluted products were detected at 280 nm.

### 2.5. Data Analysis

Microsoft Excel 2019 and GraphPad Prism 8.0 were used for analyzing and plotting data. A one-way ANOVA was performed to detect significant differences in mean values among groups, while a Pearson correlation coefficient analysis was conducted to assess the relationship between diuresis and the concentrations of the main functional components.

## 3. Results

### 3.1. Effect of Bean Extracts on Mouse Urine Output

**Figure 1:**
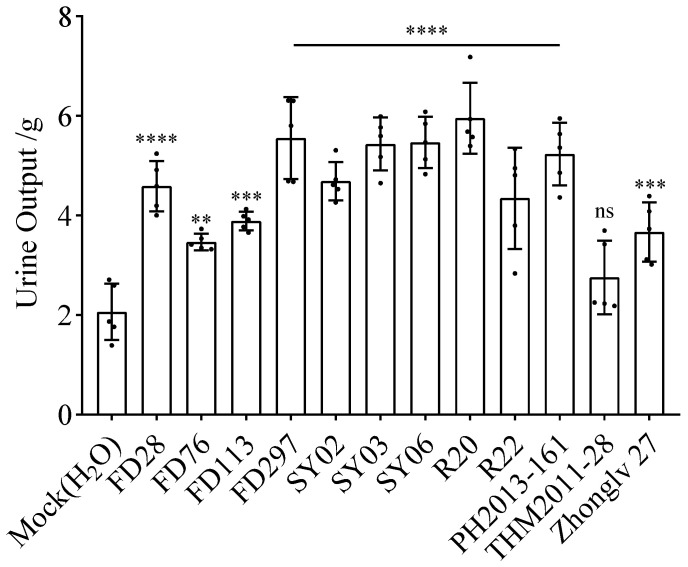
Average urine output of mice at 7 days after gavage. Asterisks indicate significant differences as determined by a one-way ANOVA: **** *p* < 0.0001, *** *p* < 0.001, and ** *p* < 0.01; ns, not significant (*p* > 0.05).

At 7 days after gavage with supernatants of different bean extracts, the average urine output was calculated. The urine output of the mock control group (2.0 g) differed significantly from that of the treatment groups, except for THM2011-28 (2.8 g). Urine output was highest for R20 (6.0 g), followed by FD297 (5.6 g), SY03 (5.4 g), and SY06 (5.5 g). Urine output was lowest for FD76 (3.4 g), followed by THM2011-28 (2.8 g) (Figure 1).

The hourly urine volume differed between the mock control group and the treatment groups. With the exception of FD76, the treatment groups had a significant increase in the urine output in the first hour after gavage (relative to the urine output for the mock control group). In the second and third hours after gavage, the urine volume decreased significantly. In the second hour, the urine volume of the FD28, FD113, FD297, SY02, SY03, SY06, R20, and PH2013-161 treatment groups differed significantly from that of the mock control group. However, in the third hour, only SY06 and R20 had a urine volume that differed significantly from that of the mock control group. Thus, the urine output of mice peaked in the first hour after gavage and then gradually decreased. During the first 3 h, the urine volume was highest in the R20 treatment group, reflecting the strong diuretic effects of this accession (Figure 1, Table 2).

### 3.2. Effect of Bean Extracts on Mouse Weight

**Figure 2:**
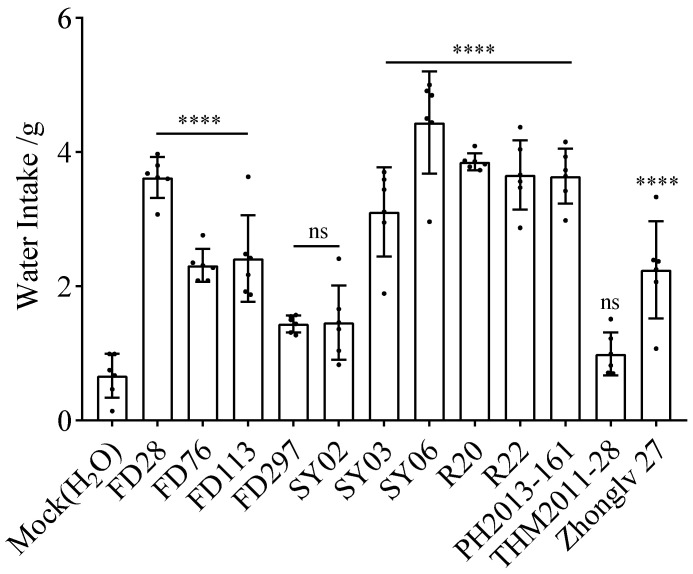
Average weight loss of mice within 7 days after gavage. Asterisks indicate significant differences as determined by a one-way ANOVA: **** *p* < 0.0001, ns, not significant (*p* > 0.05).

The comparison with the mock control group revealed the weight of the mice in all treatment groups decreased after gavage (Figure 2), although the decrease was not significant for FD297, SY02, and THM2011-28. According to the average values, the weight loss after gavage was highest for the mice in the rice bean treatment groups. More specifically, the weight loss was highest for SY 06 (decreased by 4.44 g), followed by R20 (decreased by 3.86 g). In contrast, the lowest weight loss after gavage was observed for FD297.

### 3.3. Water Intake after Gavage with Bean Extracts

**Figure 3:**
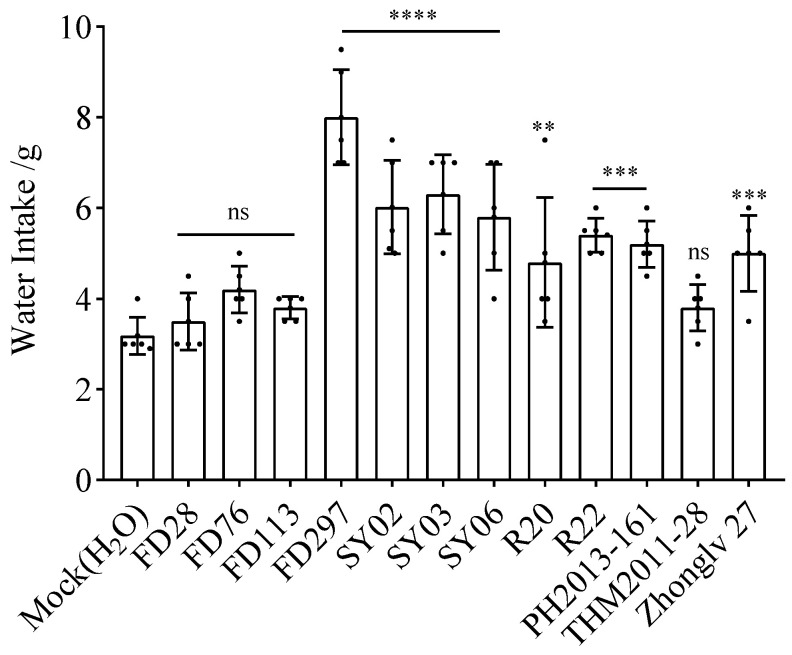
Seven-day average water intake of mice after gavage. Asterisks indicate significant differences as determined by a one-way ANOVA: **** *p* < 0.0001, *** *p* < 0.001,** *p* < 0.01; ns, not significant (*p* > 0.05).

Water intake after gavage was higher for the mice treated with rice bean extracts than for the mice treated with adzuki bean or mung bean extracts. The 7-day average water intake after gavage was significantly higher for the treatment groups, except for FD28, FD113, and PH2013-161, than for the mock control group. The water intake after gavage was highest for FD297 (8.00 g), followed by SY03 (6.30 g), R20 (6.02 g), and R22 (5.40 g). The lowest water intake was observed for FD28 (3.50 g) (Figure 3).

### 3.4. Correlation between Seed Traits and Rice Bean Diuretic Effects

**Figure 4:**
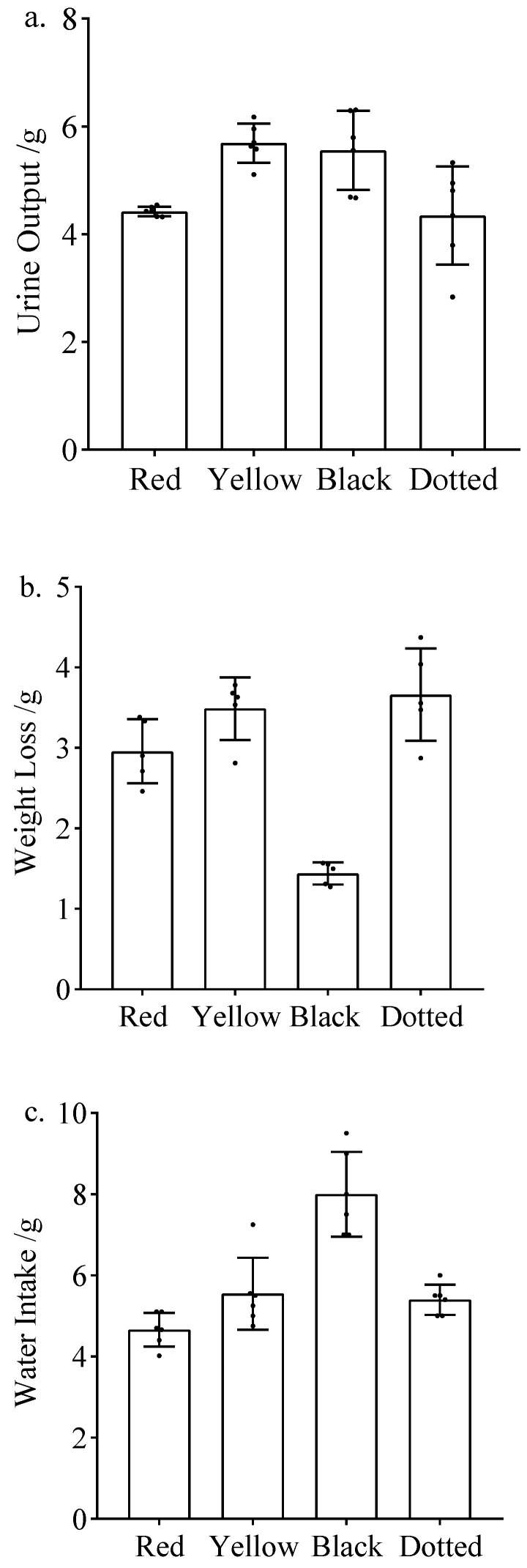
Diuretic effects of rice bean accessions with different seed colors. (**a**) Seven-day average urine output of mice after gavage. (**b**) Seven-day average weight loss of mice after gavage. (**c**) Seven-day average water intake of mice after gavage.

The seeds of the tested rice bean accessions were red (FD28, FD76, FD113, SY02, and SY06), yellow (SY03 and R20), black (FD297), and dotted (R22). The correlation analysis of the rice bean seed color and the diuretic-related indices revealed the urine output was highest for the mice gavaged with yellow seed extracts(5.8 g), followed by the mice gavaged with the black seed extract (5.6 g) (Figure 4a). In terms of water intake, the highest value was observed for the mice gavaged with the black seed extract (7.71 g), followed by the mice gavaged with the yellow seed extract (6.67 g). Notably, the mice gavaged with red seed extracts had the lowest urine output and water intake. The weight loss was highest for the mice gavaged with yellow seed extracts (3.39 g), followed by the mice gavaged with the red seed extracts (2.89 g) (Figure 4b,c).

**Figure 5:**
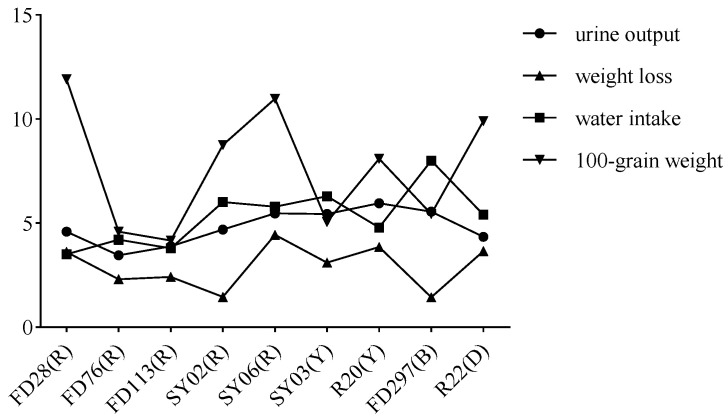
Rice bean hundred-grain weight and trends in urine output, weight loss, and water intake of mice after gavage. Letters in parentheses indicate seed color: R, red; Y, yellow; B, black; and D, dotted.

According to the correlation analysis, there were no obvious correlations between rice bean seed size and urine output (R = 0.20), water intake (R = −0.15), or weight loss (R = 0.59) (Figure 5).

### 3.5. Polyphenol Contents of Dry Seeds

Bean accessions were examined in terms of the abundance of eight secondary metabolites. The rutin concentration was highest in rice bean, with an average of 232.2 µg/g, which was considerably higher than the corresponding concentration in adzuki bean (37.4 µg/g). The rutin content in mung bean was much lower (7.6 µg/g). Of the rice bean accessions, FD113 had the highest rutin concentration (311.8 µg/g), followed by the two accessions with yellow seeds (246.3 and 248.8 µg/g). The average catechin concentrations in rice bean and adzuki bean were 64.4 and 108.2 µg/g, respectively. The vitexin and isovitexin concentrations were much higher in mung bean (849.2 and 683.5 µg/g, respectively) than in rice bean (21.6 and 17.9 µg/g, respectively) or adzuki bean (13.4 and 17.0 µg/g, respectively). The concentrations of the other four analyzed metabolites (gallic acid, chlorogenic acid, caffeic acid, and genistein) were relatively low (less than 5 µg/g) in the three legumes.

**Figure 6:**
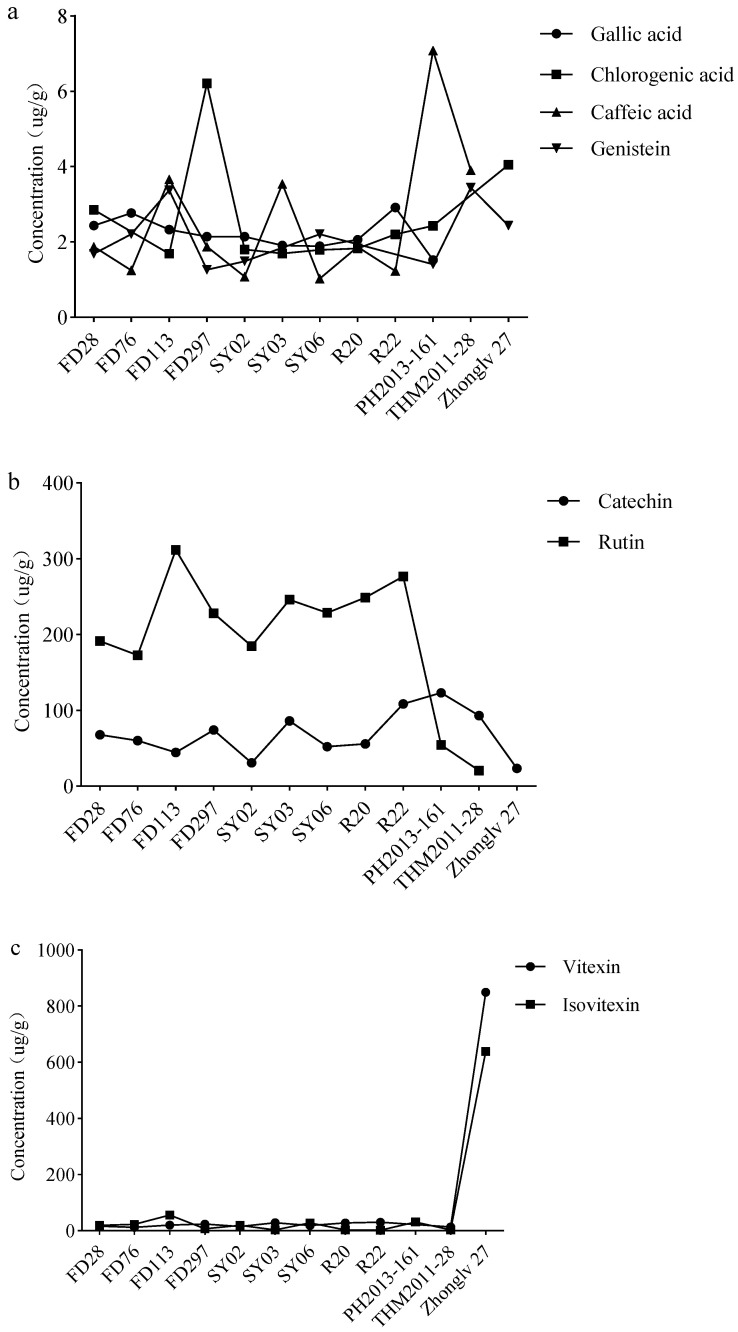
Concentrations of the main functional components of different legumes. (**a**) Gallic acid, chlorogenic acid, caffeic acid, and genistein concentrations. (**b**) Catechin and rutin concentrations. (**c**) Vitexin and isovitexin concentrations.

The polyphenol contents varied among the rice bean accessions. For example, the rutin concentration ranged from 172.7 µg/g (FD76) to 311.8 µg/g (FD113), whereas the catechin concentration ranged from 30.9 µg/g (SY02) to 108.6 µg/g (R22). Overall, there were no obvious correlations between the polyphenol (rutin and catechin) concentrations and rice bean seed weight, but there were some trends in the polyphenol (rutin and catechin) concentrations of rice bean accessions with differing seed colors. More specifically, if FD113 (red seeds) was not considered, the rutin concentration was highest in the accessions with yellow seeds (246.3 and 248.8 µg/g in SY03 and R20, respectively) and the accession (R22) with dotted seeds (276.8 µg/g). Additionally, the accessions with the three highest catechin concentrations were R22 (108.6 µg/g), SY03 (86.0 µg/g), and FD297 (74.2 µg/g) (Figure 6).

### 3.6. Correlation between Polyphenol Concentrations and the Diuresis-Related Indices

**Figure 7:**
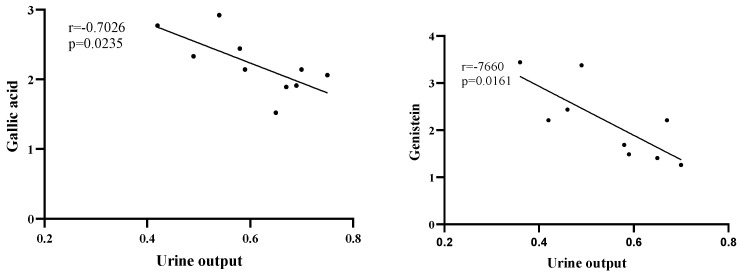
Relationship between urine output and gallic acid and genistein contents.

Correlation analyses were conducted to determine the relationships between diuresis and polyphenol concentrations. Urine output was significantly negatively correlated with the gallic acid (R = −0.70) and genistein (R = −0.75) concentrations (Figure 7). Urine output had a relatively weak negative correlation with caffeic acid (R = −0.05), vitexin (R = −0.26), and isovitexin (R = −0.30) concentrations, but a weakly positive correlation with chlorogenic (R = 0.02), catechin (R = 0.08), and rutin (R = 0.37) concentrations. Conversely, weight loss and water intake were not correlated with polyphenols, except for chlorogenic acid (R = −0.52 and 0.51, respectively) and genistein (R = −0.62 for the correlation with water intake) (Table 3), but these correlations were not significant.

## 4. Discussion

Diuretics are important for treating many diseases, including hypertension, kidney disorders, and acute heart failure [26,27,28,29]. However, because chemically synthesized diuretics used in clinical practice have potential side effects [30,31,32], there is a need for natural diuretics. In traditional Chinese medicine, rice bean has been used as a natural diuretic. Numerous studies on rice bean in the past few decades have focused on its agronomic traits [33,34,35] and genetic markers [36,37,38,39], but there has been relatively little research on its medicinal value (e.g., using animal models to confirm its diuretic effect). There are several reasons for mouse models that are often used to study diuresis (e.g., dosage, simplicity, and reliability) [40,41]. In the current study, we explored the diuretic effects of rice bean accessions using a mouse model. The results revealed differences in the diuretic effects of the examined legume species, but all three species had better diuretic effects than the mock control. Notably, rice bean had a greater diuretic effect than adzuki bean or mung bean. For all treatment groups, urine volume peaked in the first hour after gavage and then tended to decrease at the subsequent time points (Table 2). These results reflect the rapid diuretic effect of legumes, but the duration of the diuretic effect is relatively short.

The weight of mice decreased after gavage, with the weight of most treatment groups differing significantly from the weight of the mock control group. However, there were no obvious differences among the effects of the three bean extracts on mouse weight, suggesting that weight loss might not be an effective index for assessing diuresis. Water intake was higher for the treatment groups than for the mock control group. For some groups, water intake gradually decreased in the 7 days after gavage, but for other groups, water intake was relatively stable, indicative of the differences in rice bean diuretic effects. Notably, the mice gavaged with FD113 had the highest water intake, but their urine volume was lower than that of the other mice. Moreover, water intake and urine volume were high for the mice gavaged with R20, implying the diuretic effect of R20 was stronger than that of the other accessions.

The mice gavaged with R20 urinated more than the mice in the other treatment groups (Figure 1, Figure 2 and Figure 3), indicating that R20 might have the strongest diuretic effect. Overall, urine output and water intake were higher for the yellow and black rice bean seed extract treatment groups than for the red rice bean seed extract treatment group (Figure 4a,c). This is inconsistent with the exclusive use of small red rice bean seeds in traditional Chinese medicine. In addition, weight loss was greater for the mice gavaged with the extracts from yellow, black, and dotted seeds than for the mice gavaged with the extracts from red seeds (Figure 4b). Moreover, there were no obvious relationships between the diuretic effects and seed size; the accessions with large seeds (FD28 and SY06) were not associated with high urine output, water intake, and weight loss (Figure 5). Although these results were not in accordance with traditional wisdom regarding the utility of rice bean, they will need to be further validated using additional accessions.

The most abundant polyphenols differed among the examined legumes (Figure 5). Vitexin and isovitexin contents were high, but only in mung bean, with concentrations that were substantially higher than those in rice bean and adzuki bean, indicating these compounds may be the main factors underlying the differential health benefits of mung bean. The rutin content is reportedly much lower in beans than in buckwheat and tea [42,43]. Nevertheless, it was the most abundant polyphenol in rice bean (184.7–311.8 µg/g), followed by catechins. The highest catechin concentration (approximately 100 µg/g), which was detected in adzuki bean, was lower than the catechin concentration in buckwheat [42,44].

Correlation analyses indicated that only gallic acid (R = −0.70) and genistein (R = −0.75) concentrations were significantly negatively correlated with the urine output, suggesting that these two polyphenols may be negative regulators of diuresis. Additionally, urine output may be a better index than weight loss or water intake for analyzing diuresis. In a previous study, gallic acid isolated from *Mimosa bimucronata* (DC.) promoted urination [41]. Moreover, the strong anti-inflammatory effect of gallic acid has been demonstrated in numerous studies [45]. Notably, there are some inconsistencies in the functional characterization of gallic acid, which may be mainly related to the combined pharmacological effects of different plant extracts [46]. A slight negative relationship between diuresis and vitexin/isovitexin concentrations was detected. This suggests mung bean, which has high vitexin and isovitexin contents, does not induce diuresis. However, there are reports describing the high vitexin uptake rate of the kidney [47,48], implying this organ may have a relatively high affinity for vitexin and isovitexin. We also determined that urine output and water intake were positively correlated with the rutin concentration, whereas weight loss was positively correlated with the catechin concentration, but these correlations were insignificant. Earlier research suggested that rutin can increase glutathione peroxidase activities in the kidney, while also decreasing malondialdehyde levels, indicative of its protective effects on the kidney [49,50,51]. As the second most abundant polyphenol in rice bean seeds, catechins may not be related to the diuretic effects of rice bean. In tea, catechins are the main polyphenols [52], which have antioxidant, anticancer, diuretic, and other medicinal effects [53,54,55]. Other studies showed that catechins can protect the kidney via their antioxidant and anti-inflammatory properties [56,57]. The results of the correlation analysis conducted in the present study imply that rutin and catechins may not mediate the diuretic effect of rice bean. Specifically, despite its high rutin and catechin concentrations, R22 had a weaker diuretic effect than R20, which has a high gallic acid content.

It is important to note that the complex structures of secondary metabolites may not be conducive to absorption in vivo. This may contribute to the discrepancies between the results of in vitro and in vivo studies or the inconsistencies between the findings of different laboratory tests. For example, the health-related effects of catechins varied among diverse trials, likely because of the low bioavailability of catechins [58]. Polyphenols in legumes are typically detected at concentrations that are much higher than the concentrations of the eight polyphenols we examined [24], and we only analyzed a few common metabolites. Thus, the present results may be used as the basis of future investigations. In addition, because the effects of many secondary metabolites are detectable even at low concentrations, future research should focus on additional secondary metabolites and systematic animal experiments.

## 5. Conclusions

On the basis of the mouse gavage test and the correlation analysis of the diuresis-related indices and polyphenol concentrations of 12 accessions of three *Vigna* species, we revealed that rice bean had the strongest diuretic effect, followed by adzuki bean. However, rice bean accessions with red seeds had smaller effects on urine output, water intake, and weight loss than rice bean accessions with yellow, black, or speckled seeds. Seed size was not correlated with the analyzed diuresis-related indices. Interestingly, the vitexin and isovitexin concentrations were much higher in mung bean than in rice bean or adzuki bean. There were no obvious relationships among water intake, weight loss, and polyphenol concentrations, except for the significant negative correlation between urine output and gallic acid and genistein concentrations. However, because of the limited number of accessions used in this study, future investigations will need to validate the results presented herein. Furthermore, systematic animal experiments will need to be conducted using additional polyphenolic compounds.

## Figures and Tables

**Table 1:** Details regarding the plant species and accessions used for gavage.

Species	Code	Color	100-Grain Weight/g
Rice bean	FD28	Red	11.92
	FD76	Red	4.59
	FD113	Red	4.17
	FD297	Black	5.44
	SY02	Red	8.76
	SY03	Yellow	5.06
	SY06	Red	10.98
	R20	Yellow	8.10
	R22	Dotted	9.90
Adzuki bean	PH2013-161	Red	13.9
	THM2011-28	Red	16.2
Mung bean	Zhonglv 27	Green	4.5

**Table 2:** Hourly urine output of mice after gavage (unit: g).

Group	1th h	2nd h	3rd h	4th h	5th h	6th h	7th h	8th h
FD28	1.54 ± 0.15 ****	0.78 ± 0.21 ***	0.35 ± 0.18	0.40 ± 0.23	0.32 ± 0.07	0.31 ± 0.03	0.35 ± 0.13	0.45 ± 0.21
FD76	0.73 ± 0.16	0.50 ± 0.09	0.29 ± 0.12	0.26 ± 0.12	0.29 ± 0.15	0.27 ± 0.19	0.31 ± 0.18	0.38 ± 0.15
FD113	1.08 ± 0.16 **	0.71 ± 0.23 **	0.23 ± 0.06	0.35 ± 0.16	0.26 ± 0.14	0.25 ± 0.13	0.29 ± 0.19	0.61 ± 0.15 *
FD297	1.56 ± 0.11 ****	0.81 ± 0.18 ***	0.25 ± 0.08	0.34 ± 0.13	0.35 ± 0.21	0.80 ± 0.40 ****	0.62 ± 0.17 **	0.81 ± 0.25 ***
SY02	1.54 ± 0.32 ****	0.69 ± 0.08 **	0.36 ± 0.16	0.22 ± 0.17	0.37 ± 0.22	0.32 ± 0.10	0.45 ± 0.10	0.62 ± 0.21 *
SY03	1.76 ± 0.34 ****	0.90 ± 0.22 ****	0.29 ± 0.09	0.36 ± 0.29	0.27 ± 0.06	0.27 ± 0.06	0.46 ± 0.21	0.67 ± 0.24 *
SY06	2.02 ± 0.58 ****	0.70 ± 0.16 **	0.42 ± 0.23 *	0.46 ± 0.12	0.37 ± 0.16	0.44 ± 0.12	0.41 ± 0.23	0.46 ± 0.27
R20	1.94 ± 0.16 ****	0.86 ± 0.19 ****	0.59 ± 0.26 ***	0.40 ± 0.27	0.37 ± 0.16	0.41 ± 0.13	0.55 ± 0.16 *	0.51 ± 0.11
R22	1.50 ± 0.30 ****	0.44 ± 0.10	0.27 ± 0.26	0.35 ± 0.12	0.32 ± 0.10	0.41 ± 0.11	0.46 ± 0.34	0.59 ± 0.25
PH2013-161	1.92 ± 0.27 ****	0.65 ± 0.18 *	0.40 ± 0.14	0.39 ± 0.09	0.28 ± 0.11	0.30 ± 0.12	0.66 ± 0.12 **	0.62 ± 0.24 *
THM2011-28	1.23 ± 0.27 ***	0.30 ± 0.14	0.14 ± 0.10	0.30 ± 0.18	0.09 ± 0.06	0.14 ± 0.06	0.17 ± 0.05	0.45 ± 0.28
Zhonglv 27	1.51 ± 0.19 ****	0.52 ± 0.15	0.26 ± 0.08	0.18 ± 0.08	0.10 ± 0.09	0.18 ± 0.07	0.19 ± 0.11	0.56 ± 0.19
Mock (H_2_O)	0.39 ± 0.17	0.31 ± 0.11	0.13 ± 0.08	0.19 ± 0.09	0.13 ± 0.09	0.19 ± 0.07	0.22 ± 0.12	0.21 ± 0.11

Asterisks indicate significant differences as determined by a two-sided Student’s *t*-test: **** *p* < 0.0001, *** *p* < 0.001, ** *p* < 0.01, and * *p* < 0.05.

**Table 3:** Correlations between mouse physiological indices and rice bean seed functional components.

	Urine Output	Weight Loss	Water Intake
Gallic acid	−0.70 *	−0.15	−0.35
Chlorogenic acid	0.02	−0.52	0.51
Catechin	0.08	0.22	0.06
Caffeic acid	−0.05	−0.01	−0.16
Rutin	0.37	0.32	0.22
Vitexin	−0.26	−0.12	−0.03
Isovitexin	−0.3	−0.14	−0.07
Genistein	−0.75 *	−0.23	−0.62

Note: Asterisks indicate significant differences as determined by a one-way ANOVA: * *p* < 0.05.

## Data Availability

The original contributions presented in the study are included in the article, further inquiries can be directed to the corresponding author.

## References

[1] Guan J., Zhang J., Gong D., Zhang Z., Yu Y., Luo G., Somta P., Hu Z., Wang S., Yuan X. (2022). Genomic Analyses of Rice Bean Landraces Reveal Adaptation and Yield Related Loci to Accelerate Breeding. Nat. Commun..

[2] Doi K., Kaga A., Tomooka N., Vaughan D.A. (2002). Molecular Phylogeny of Genus Vigna Subgenus Ceratotropis Based on rDNA ITS and atpB-rbcL Intergenic Spacer of cpDNA Sequences. Genetica.

[3] Seehalak W., Tomooka N., Waranyuwat A., Thipyapong P., Laosuwan P., Kaga A., Vaughan D.A. (2006). Genetic Diversity of the Vigna Germplasm from Thailand and Neighboring Regions Revealed by AFLP Analysis. Genet. Resour. Crop Evol..

[4] Pattanayak A., Roy S., Sood S., Iangrai B., Banerjee A., Gupta S., Joshi D.C. (2019). Rice Bean: A Lesser Known Pulse with Well-Recognized Potential. Planta.

[5] Bhardwaj N., Kaur J., Anjali, Sharma P. (2021). Ricebean. The Beans and the Peas.

[6] Asha R., Koundinya A., Das A., Chattopadhyay S. (2019). A Review on an Underutilised Multipurpose Legume: Rice Bean. Acta Hortic..

[7] Joshi K.D., Bhandari B., Gautam R., Bajracharya J., Hollington P. (2008). Ricebean: A Multipurpose Underutilised Legume. New Crops and Uses: Their Role in a Rapidly Changing World.

[8] Bhanu A.N., Singh M.N., Srivastava K. (2018). Efficient Hybridization Procedure for Better Pod Setting in Inter-Specific Crosses Involving Vigna Species. Adv. Plants Agric. Res..

[9] Kashiwaba K., Tomooka N., Kaga A., Han O.K., Vaughan D.A. (2003). Characterization of Resistance to Three Bruchid Species (*Callosobruchus* Spp., Coleoptera, Bruchidae) in Cultivated Rice Bean (*Vigna umbellata*). J. Econ. Entomol..

[10] Kaur H., Gill R.S., Kaur R. (2019). Correlation between Biophysical Seed Characteristics of Rice Bean, Vigna Umbellata (Fabaceae: Faboideae: Phaseoleae) and the Development of Callosobruchus Maculatus (Coleoptera: Chrysomelidae: Bruchinae). J. Stored Prod. Res..

[11] Pavithravani B.V., Gowda R., Bhanuprakash K., Ramesh S., Rao M.A., Subramanya S., Gireesh C. (2013). Biochemical Components: An Index of Bruchid Resistance in Rice Bean [*Vigna umbellata* (Thunb.) Ohwi and Ohashi]. Legume Res..

[12] Sudha M., Anusuya P., Mahadev N.G., Karthikeyan A., Nagarajan P., Raveendran M., Senthil N., Pandiyan M., Angappan K., Balasubramanian P. (2013). Molecular Studies on Mungbean (*Vigna radiata* (L.) Wilczek) and Ricebean (*Vigna umbellata* (Thunb.)) Interspecific Hybridisation for Mungbean Yellow Mosaic Virus Resistance and Development of Species-Specific SCAR Marker for Ricebean. Arch. Phytopathol. Plant Prot..

[13] Atta K., Pal A.K., Jana K. (2021). Effects of Salinity, Drought and Heavy Metal Stress during Seed Germination Stage in Ricebean [*Vigna umbellata* (Thunb.) Ohwi and Ohashi]. Plant Physiol. Rep..

[14] Wanek W., Richter A. (2010). Biosynthesis and Accumulation of D-Ononitol in *Vigna umbellata* in Response to Drought Stress. Physiol. Plant..

[15] Dhillon P.K., Tanwar B. (2018). Rice Bean: A Healthy and Cost-Effective Alternative for Crop and Food Diversity. Food Secur..

[16] Wang L.X., Cheng X.Z., Wang S.H. (2014). Genetic Diversity Analysis and a Core Collection Construction of Rice Bean (*Vigna umbellata*) in China. J. Plant Genet. Resour..

[17] Arora R.K., Chandel K.P.S., Joshi B.S., Pant K.C. (1980). Rice Bean: Tribal Pulse of Eastern India. Econ. Bot..

[18] Mohan V., Janardhanan K. (1994). Chemical Composition and Nutritional Evaluation of Raw Seeds of Six Ricebean Varieties. J. Indian Bot. Soc..

[19] Katoch R. (2013). Nutritional Potential of Rice Bean (*Vigna umbellata*): An Underutilized Legume. J. Food Sci..

[20] Bepary R.H., Wadikar D.D., Neog S.B., Patki E.P. (2017). Studies on Physico-Chemical and Cooking Characteristics of Rice Bean Varieties Grown in NE Region of India. J. Food Sci. Technol..

[21] Rodriguez M.S., Mendoza E.M.T. (1991). Nutritional Assessment of Seed Proteins in Rice Bean [*Vigna umbellata* (Thumb.) Ohwi and Ohashi. Plant Foods Hum. Nutr..

[22] Cui G., Chang Y. (2014). Red bean and mung bean with homologous medicinal and food sources. Cap. Food Med..

[23] Chau C.F., Cheung P.C. (1999). Effects of the Physico-Chemical Properties of Three Legume Fibers on Cholesterol Absorption in Hamsters. Nutr. Res..

[24] Yao Y., Cheng X.Z., Wang L.X., Wang S.H., Ren G.X. (2011). Biological Potential of Sixteen Legumes in China. Int. J. Mol. Sci..

[25] Hong J., Bai B., Bi Y. (2020). Study on Antioxidant Activity of Total Polyphenols of Phaseolus Calcaratus Roxb. Guangdong Chem. Ind..

[26] Yu Y., Hu D., Liu J., Wu C., Sun Y., Lang M., Han X., Kang D., Min J.Z., Cui H. (2024). Constituents of Chimaphila Japonica and Their Diuretic Activity. Mol. Basel Switz..

[27] Ngamlai E.V., Pradhan R.B., Lalbiaknii P.C., Ralte V., Lalnunmawia F., Vanlalhluna P.C., Mehta S.K. (2024). Diuretic Activity Evaluation and Chemical Composition Analysis of Hedyotis Scandens Extract from Mizoram, India, in Rat Models. J. Ethnopharmacol..

[28] Boockvar K.S., Song W., Lee S., Intrator O. (2020). Comparing Outcomes Between Thiazide Diuretics and Other First-Line Antihypertensive Drugs in Long-Term Nursing Home Residents. Clin. Ther..

[29] Cox Z.L., Sarrell B.A., Cella M.K., Tucker B., Arroyo J.P., Umanath K., Tidwell W., Guide A., Testani J.M., Lewis J.B. (2022). Multinephron Segment Diuretic Therapy to Overcome Diuretic Resistance in Acute Heart Failure: A Single-Center Experience. J. Card. Fail..

[30] Gupta S., Neyses L. (2005). Diuretic Usage in Heart Failure: A Continuing Conundrum in 2005. Eur. Heart J..

[31] He H., Sui Y., Yu X., Luo G., Xue J., Yang W., Long Y. (2024). Potential Low Toxic Alternative for Na-Cl Cotransporter Inhibition: A Diuretic Effect and Mechanism Study of Pyrrosia Petiolosa. Ann. Pharm. Françaises.

[32] Titko T., Perekhoda L., Drapak I., Tsapko Y. (2020). Modern Trends in Diuretics Development. Eur. J. Med. Chem..

[33] Gupta S., Kozak M., Sahay G., Durrai A.A., Mitra J., Verma M.R., Pattanayak A., Thongbam P.D., Das A. (2009). Genetic Parameters of Selection and Stability and Identification of Divergent Parents for Hybridization in Rice Bean (*Vigna umbellata* Thunb. (Ohwi and Ohashi) in India. J. Agric. Sci.-Camb..

[34] Singh G., Singh M., Dhiman K.R. (1992). Correlation and Path Analysis in Ricebean under Mid Altitude Conditions. Crop Improv..

[35] Singh M.R.K., Chakravarti D., Singh N.B. (1997). Genetic Variability, Correlation and Path Analysis in Rice Bean (*Vigna umbellata* (Thunb.) Ohwi and Ohashi) Cultivars of Manipur. Indian J. Hill Farming.

[36] Isemura T., Kaga A., Tomooka N., Shimizu T., Vaughan D.A. (2010). The Genetics of Domestication of Rice Bean, *Vigna umbellata*. Ann. Bot..

[37] Chen H., Chen X., Tian J., Yang Y., Liu Z., Hao X., Wang L., Wang S., Liang J., Zhang L. (2016). Development of Gene-Based SSR Markers in Rice Bean (*Vigna umbellata* L.) Based on Transcriptome Data. PLoS ONE.

[38] Muthusamy S., Kanagarajan S., Ponnusamy S. (2008). Efficiency of RAPD and ISSR Markers System in Accessing Genetic Variation of Rice Bean (*Vigna umbellata*) Landraces. Electron. J. Biotechnol..

[39] Wang L., Kim K.D., Gao D., Chen H., Wang S., Lee S.H., Jackson S.A., Cheng X. (2016). Analysis of Simple Sequence Repeats in Rice Bean (*Vigna umbellata*) Using an SSR-Enriched Library. Crop J..

[40] Kurkin V.A., Zaitseva E.N., Kurkina A.V., Dubishchev A.V., Pravdivtseva O.E. (2015). Comparative Study of Diuretic Activity of Hydroalcoholic Extracts from Medicinal Plants Containing Flavonoids. Bull. Exp. Biol. Med..

[41] Schlickmann F., Boeing T., Mariano L.N.B., da Silva R.D.C.M.V.D.A.F., da Silva L.M., de Andrade S.F., de Souza P., Cechinel-Filho V. (2018). Gallic Acid, a Phenolic Compound Isolated from Mimosa Bimucronata (DC.) Kuntze Leaves, Induces Diuresis and Saluresis in Rats. Naunyn. Schmiedebergs Arch. Pharmacol..

[42] Liu C., Chen Y., Hu Y. (2022). Study on the Content and Antioxidant Effect of Catechin and Rutin in Different Tea. Chin. J. Public Health Eng..

[43] Mu Z., Tian X., Zhou J., Shi Y. (2015). The Determination of Rutin Content in Buckwheat by HPLC. J. Shanxi Agric. Sci..

[44] Zhang H., Ma R., Liu Y., Wei Y., Dai L. (2018). Simultaneous determination of 4 components in Fagopyrum dibotrys by HPLC. Cent. South Pharm..

[45] Thomford N.E., Senthebane D.A., Rowe A., Munro D., Seele P., Maroyi A., Dzobo K. (2018). Natural Products for Drug Discovery in the 21st Century: Innovations for Novel Drug Discovery. Int. J. Mol. Sci..

[46] Tong C., Liu X. (2007). Pharmacokinetics of vitexin in rats. J. China Pharm. Univ..

[47] Huang Y., Zhang Z., Zheng L., He F., Shi L., Lan Y. (2012). UPLC—MS /MS tissue distribution of four bioactive flavonoids in *Polygonum orientale* L. extract. Chin. J. Pharm. Anal..

[48] Feng X., Chen Y., Cai C., Jiang J., Shi G. (2015). Diuretic effect of different extracted parts of grape on mice. J. Shantou Univ. Med. Coll..

[49] Abarikwu S.O., Njoku R.C., Lawrence C.J., Charles I.A., Ikewuchi J.C. (2017). Rutin Ameliorates Oxidative Stress and Preserves Hepatic and Renal Functions Following Exposure to Cadmium and Ethanol. Pharm. Biol..

[50] Enogieru A.B., Haylett W., Hiss D.C., Bardien S., Ekpo O.E. (2018). Rutin as a Potent Antioxidant: Implications for Neurodegenerative Disorders. Oxid. Med. Cell. Longev..

[51] Ma X., Zhou Y., Wang L. (2022). Protective effect of rutin on kidney damage induced by high sugar and fat diet in mice. J. Bengbu Med. Coll..

[52] Fu W., Yang J., Hong Z., Gui C., Song J., Zhang L. (1999). A Primary Study on General Pharmacologic Action of Tea Polyphenols. Acta Acad. Med. Wannan.

[53] Khan N., Mukhtar H. (2018). Tea Polyphenols in Promotion of Human Health. Nutrients.

[54] Yang C.S., Wang X., Lu G., Picinich S.C. (2009). Cancer Prevention by Tea: Animal Studies, Molecular Mechanisms and Human Relevance. Nat. Rev. Cancer.

[55] Li Y., Jiang C., Wang X. (2002). Research progress in the biological activity and pharmacology of tea polyphenols. J. Anhui Tcm Coll..

[56] Tian L., Liu Z., Song L., Yao Y. (2010). Research progress of catechins in treating kidney disease. Guangdong Tea.

[57] Wongmekiat O., Peerapanyasut W., Kobroob A. (2018). Catechin Supplementation Prevents Kidney Damage in Rats Repeatedly Exposed to Cadmium through Mitochondrial Protection. Naunyn. Schmiedebergs Arch. Pharmacol..

[58] Cai Z.-Y., Li X.-M., Liang J.-P., Xiang L.-P., Wang K.-R., Shi Y.-L., Yang R., Shi M., Ye J.-H., Lu J.-L. (2018). Bioavailability of Tea Catechins and Its Improvement. Mol. Basel Switz..

